# Study on the Photocatalytic Properties of Metal–Organic Framework-Derived C-, N-Co-Doped ZnO

**DOI:** 10.3390/ma17040855

**Published:** 2024-02-12

**Authors:** Su Fu, Wenkui Xi, Jinlong Ren, Hangxin Wei, Wen Sun

**Affiliations:** 1 School of Mechanical Engineering, Xi’an Shiyou University, Xi’an 710065, China; fs020824@163.com (S.F.);; 2 School of Materials Science and Engineering, Lanzhou University of Technology, Lanzhou 730050, China

**Keywords:** metal–organic framework (MOF), ZIF-8, ZnO, C-, N-doping, photocatalysis

## Abstract

In this study, C- and N-co-doped ZnO photocatalysts were prepared through pyrolysis using metal–organic frameworks (MOFs) as precursor materials. The crystal structure, morphology, and surface chemical composition of the samples were characterised via X-ray diffraction (XRD), scanning electron microscopy (SEM), and X-ray photoelectron spectroscopy (XPS). Their activities in photocatalytic reactions were also evaluated through photocatalytic experiments. The results show that C-, N-co-doped ZnO has a high specific surface area, which is favourable for a photocatalytic reaction. Meanwhile, C-, N-doping can effectively modulate the energy band structure of ZnO, broaden its light absorption range, and improve the separation efficiency of photogenerated electron–hole pairs. The photocatalytic experiments show that the C/N-ZnO-500 samples, which have the optimal photocatalytic performances, have improved performances of 50% and 35%, respectively, compared with those of the blank control group and the ZIF-8 samples. The preparation of ZnO materials with a morphology change and doping using metal frameworks as precursors provides a new idea for designing efficient photocatalysts.

## 1. Introduction

In recent years, due to increasingly serious environmental pollution and the energy crisis, photocatalytic technology, as a green, environmentally friendly, and highly efficient technology, has received extensive attention and research [1,2]. As one of the most important third-generation semiconductor materials, ZnO is a wide-band-gap semiconductor material with a direct band gap and a band width of 3.37 eV. It has the advantages of being nontoxic, cheap, with good stability, and excellent optical and electrical properties, etc. As an important semiconductor photocatalytic material, it has been widely used in the fields of photocatalytic water decomposition and the photocatalytic degradation of organic pollutants. However, due to their small specific surface area, low light absorption efficiency, and large forbidden bandwidth, conventional ZnO materials limit the further improvement of photocatalytic activity [3,4]. To solve this problem, various methods, such as elemental doping [5,6], morphology control [7,8], heterostructure building [9,10], and noble metal nanoparticle modification [11,12] have been investigated to improve the photocatalytic performance of ZnO and thus further enhance its photocatalytic efficiency. Among them, the control of ZnO morphology and elemental doping can not only increase the specific surface area, but also form defects and impurity energy levels, which can regulate the forbidden bandwidth, improve the efficiency and probability of electron leaps and, at the same time, inhibit the recombination of photogenerated carriers and increase the photocatalytic activity of ZnO, which can improve the photocatalytic properties of ZnO effectively [13,14]. For example, Yan Lin et al. [15] successfully synthesised one-dimensional scale-like zinc oxide (1D ZnO) and three-dimensional flower-like zinc oxide (3D ZnO) by treating ZnO with hydrogen. It was found that the photocurrent of one-dimensional zinc oxide and three-dimensional zinc oxide under visible light irradiation after hydrogen treatment increased by 120% and 400%, respectively. In addition, the degradation of methylene blue by three-dimensional ZnO is enhanced by 500%, and that by one-dimensional ZnO is enhanced by 130%. Xuehua Zhang et al. [16] synthesised ZnO materials with different morphologies, including ZnO nanorods (R-ZnO), ZnO nanopillars (C-ZnO), and multistage microspheres consisting of ZnO nanosheets (M-ZnO) by adjusting the concentration of the growth solution using a two-step hydrothermal method. By observing the degradation of methyl orange, the photocatalytic efficiency of the M-ZnO samples was found to be as high as 79%. These results indicate that morphology control can greatly improve photocatalytic performance. Md. Rashid Al-Mamun et al. [17] prepared metal (Cu and Ni)-doped ZnO nanocomposites and showed that the addition of dopant nickel to the ZnO lattice decreased the band gap energy from 3.44 eV to 3.16 eV. After UV irradiation for 180 min, the photocatalytic activity of ZnO, Cu/ZnO, Ni/ZnO, and Cu/Ni/ZnO photocatalysts for the degradation of methyl orange was 76.31%, 81.95%, 89.30%, and 83.39%, respectively, and the Ni/ZnO nanocomposites showed excellent reusability, which was 81% after four cycles of continuous use. The increase in photocatalytic efficiency was attributed to the reduction of the photocatalyst band gap energy and the crystallite size.

A metal–organic framework (MOF), a novel porous material, consists of metal clusters and organic ligands. It features a high specific surface area, tunable pore structure, and functionalised surface properties, and has received extensive attention owing to its high porosity and wide applications in adsorption, catalysis, and other fields [18,19]. Inorganic nanomaterials derived from MOFs inherit the porous nature and specific elemental composition of the MOFs. This allows for the synthesising of nanomaterials with specific functions [20]. Additionally, the inherited traits provide an ideal foundation for the preparation of photocatalysts with specific structures and properties. Zeolite imidazolium skeleton-8 (ZIF-8) is a synthetic MOF characterised by a large specific surface area, diverse structure, adjustable pore size, easy design, and the controllability of pore surface properties. Additionally, the derivatisation of MOFs provides a novel method for fabricating C-, N-co-doped ZnO [21,22]. For example, Zheao Huang et al. [23] explored the tunable nitrogen configuration in the sample N-ZnO@NC by controlling the thermal conversion of ZIF-8. It was shown that the ZnO and nitrogen-doped carbon in N-ZnO@NC were connected through C-N-Zn bonds, which improved the charge separation efficiency and became the main reason for its excellent photocatalytic performance. DFT calculation shows that the pyridine-N configuration in MOF-derived materials is the main factor which improves performance.

Although many studies have reported the preparation of ZnO using a metal–organic framework, only single C- or N-element-doping is described. However, in the metal–organic framework, there are both C and N elements, which makes doping inevitable in the process of preparing ZnO by calcination. In order to understand the doping situation and the mechanism of C and N elements in ZnO, and to develop efficient and stable photocatalytic materials, it is necessary to deeply understand the photocatalytic mechanism of ZnO materials and provide a theoretical and experimental basis for the design and application of photocatalytic materials. In this study, MOFs were used as a precursor material to synthesise C-, N-co-doped ZnO photocatalysts through a calcination method, and the structures and optical properties of the photocatalysts were characterised. Finally, the activity of the catalysts in photocatalysis and the influence of impurity energy levels on the photocatalytic performance of ZnO were investigated.

## 2. Materials and Methods

### 2.1. Preparation of ZIF-8 Rhombic Dodecahedron

ZIF-8 is a synthetic material of MOFs formed through the in situ self-assembly of zinc ions and dimethylimidazole molecules. First, 3 mmol of Zn(NO_3_)_3_-6H_2_O and 12 mmol of dimethylimidazole (C_4_H_6_N) were dissolved in 25 mL of methanol solution and, after ultrasonication for 15 min, the two solutions were mixed with magnetic continuous stirring for 2 h and then left to age at room temperature for 24 h. The resulting white powder was washed three times with anhydrous methanol and then dried at 60 °C for 12 h to obtain ZIF-8.

### 2.2. Preparation of C-, N-Co-Doped ZnO Nano-Rhombic Dodecahedron

**Figure 1:**
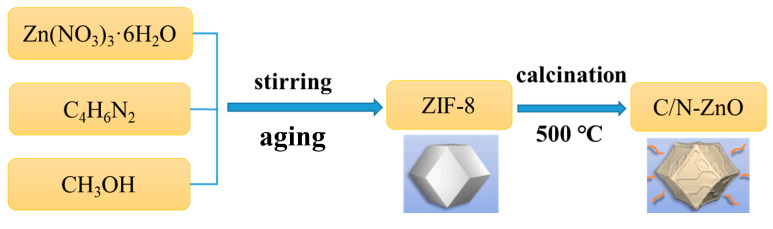
Flowchart of the preparation of ZIF-8 and C/N-ZnO.

As shown in Figure 1, C-, N-co-doped ZnO nanoparticles with ZIF-8 as the precursor were prepared via a high-temperature calcination method. The synthesised ZIF-8 powder was placed in a ceramic crucible and subsequently transferred to a muffle furnace. It was then calcined for 2 h at a heating rate of 2 °C/min to reach temperatures of 300 °C, 400 °C, 500 °C, and 600 °C, respectively. The resulting powders were labelled ZIF-8-300, C/N-ZnO-400, C/N-ZnO-500, and C/N-ZnO-600, respectively.

### 2.3. Photocatalytic Testing

The photocatalytic degradation experiment on the MO solution was carried out under simulated sunlight irradiation under a xenon lamp to study the photocatalytic performance of the sample. The wavelength of the light source was between 200 nm and 800 nm and no filter was placed. Firstly, 0.1 g of the sample was placed into a beaker containing 40 mL of the 30 mg/L MO solution, and was then irradiated under a xenon lamp with constant magnetic stirring to ensure that the catalyst would not precipitate and to maintain good contact with the target degradation reagents. The distance between the surface of the MO solution and the nozzle of the xenon lamp was controlled to 10 cm, and the concentration of the solution was tested at 5 min intervals. The change in solution concentration during the photocatalytic process was tracked by the change in the maximum absorbance of the MO solution at a 465 nm wavelength. 

### 2.4. Electrochemical Testing

A three-electrode system was used to test the electrochemical impedance of the samples, with a 0.5 mol/L Na_2_SO_4_ solution, a saturated calomel electrode, and a platinum electrode as the electrolyte, reference electrode, and counter electrode, respectively. The conductive glass was uniformly coated with a film prepared from the sample as the working electrode.

### 2.5. Materials Characterisation

The structural composition of the sample was analysed with an X-ray diffractometer (XRD, D8 Advane), and the elemental composition of the sample was tested with a cold-field-emission scanning electron microscope and its own energy dispersion spectrometer (FESEM, EDX, JMS-67005). Fluorescence spectrophotometers (PL, F97 Pro) and ultraviolet-visible diffuse reflection spectrophotometers (UV-Vis DRS, U-3900H) were used to study the optical properties of the samples. The electrochemical characteristics of the samples were studied using the electrochemical workstation (CHI660D). The surface element binding states of the samples were analysed through X-ray photoelectron spectroscopy (XPS, AXIS SUPRA).

## 3. Results

**Figure 2:**
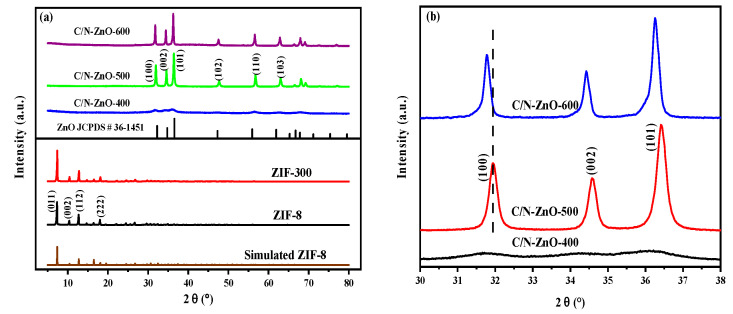
(**a**) XRD and (**b**) zoomed-in XRD spectra of different samples.

Figure 2a shows the X-ray diffraction (XRD) spectra of the as-prepared samples. The peak positions of ZIF-8 matched with the simulated peak curve results, indicating the successful preparation of ZIF-8. Peaks at 2θ of 7.39°, 10.40°, 12.77°, 14.75°, 16.50°, 18.08°, 19.53°, and 26.75° corresponded to the (110), (200), (211), (220), (013), (222), (123), and (134) crystal planes of ZIF-8, respectively [24,25]. Compared with ZIF-8, ZIF-8-300 exhibited no significant change in the XRD peak, indicating that ZIF-8 featured good thermal stability at 300 °C and that no phase transition occurred. The ZnO-400 sample featured a weak characteristic diffraction peak of ZnO, while the characteristic peak of ZIF-8 was nonexistent, which indicates that the transition from ZIF-8 to ZnO started to occur when the temperature reached 400 °C and that, at this time, ZnO was in a partially amorphous state with poor crystallinity, low peak intensity, and broadening. When the temperature reached 500 °C and 600 °C, the characteristic peak intensity of ZnO was gradually enhanced and sharpened, indicating that the increase in temperature favoured the growth of ZnO grains and crystallinity. Additionally, the diffraction peaks of ZnO were slightly shifted toward a smaller angle with the increase in calcination temperature. This is attributable to the thermal decomposition of the ZIF-8 precursor, causing the incorporation of C and N into the ZnO lattice. This resulted in lattice distortion and alterations in the crystal spacing.

**Figure 3:**
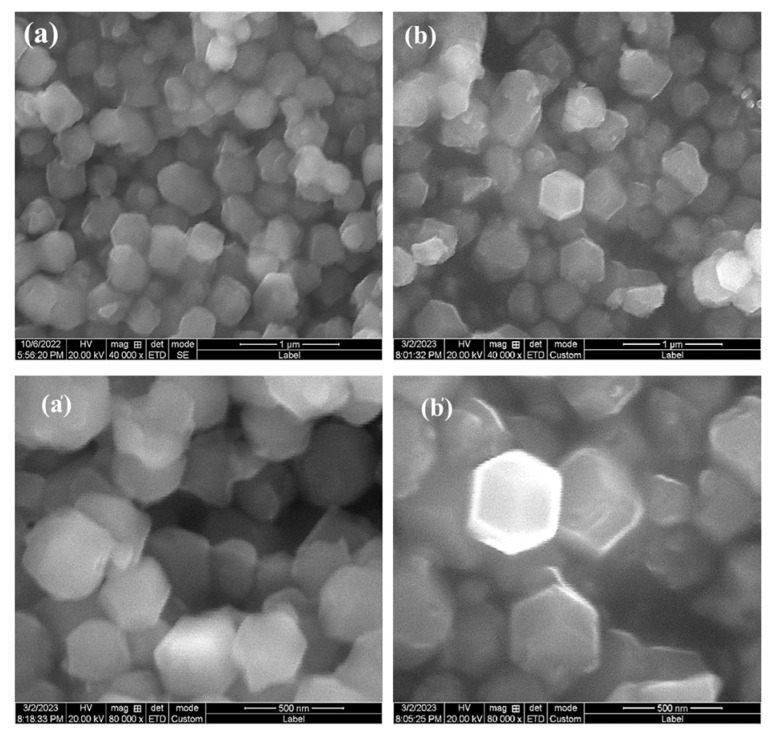
SEM images at different magnifications: (**a**,**a′**) ZIF-8; (**b**,**b′**) ZIF-8-300.

Figure 3 shows the SEM images of ZIF-8 and ZIF-8-300 samples at different magnifications, and it can be seen from Figure 3a,a′ that ZIF-8 is a dodecahedral particle of uniform size and uniform distribution, and it indicates smoother and contoured edges. As can be seen in Figure 3b,b′, the contours of the particles of the ZIF-8-300 sample became clear and the morphology did not change significantly compared to the ZIF-8 sample which, together with the XRD results, indicates the successful preparation of ZIF-8 and the absence of a phase transition of ZIF-8 when the sintering temperature was 300 °C.

**Figure 4:**
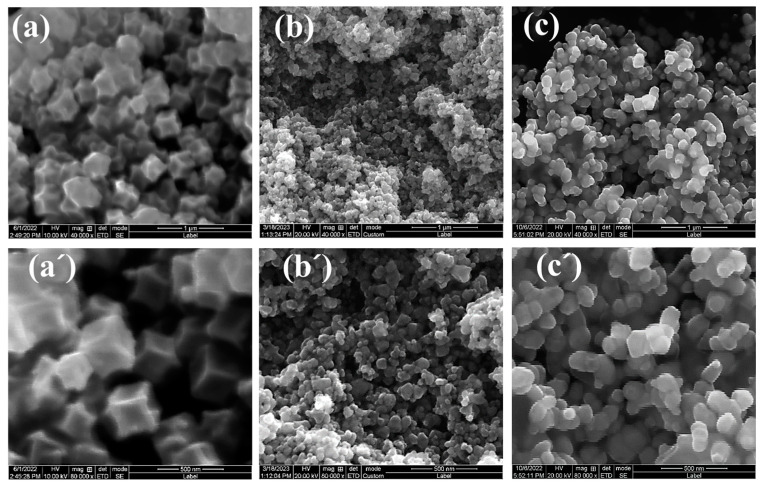
SEM images at different magnifications: (**a**,**a′**) C/N-ZnO-400; (**b**,**b′**) C/N-ZnO-500; (**c**,**c′**) C/N-ZnO-600.

Figure 4 shows the SEM images of C/N-ZnO-x. According to Figure 4a,a′, C/N-ZnO-400 exhibited a hexahedral square-block granular morphology, with each face displaying a noticeable concave feature. A significant morphological change occurred compared with ZIF-8-300, indicating the transition from ZIF-8 to ZnO upon calcination at 400 °C. From Figure 4b,b′,c,c′, it can be seen that the morphology of C/N-ZnO-500 and C/N-ZnO-600 samples is agglomerated irregular particles, that the edge contour becomes blurred, the morphology of ZnO changes gradually with the increase in calcination temperature, and the size of the particles decreases and then increases. Notably, C/N-ZnO-500 exhibited the smallest particle size. According to the SEM and XRD results, the crystallinity of ZnO gradually increased with the increase in calcination temperature.

**Figure 5:**
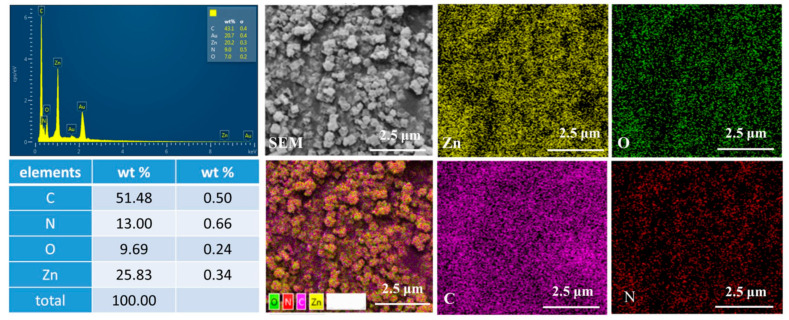
EDS element-mapping and SEM images of C/N-ZnO-500.

Figure 5 presents the energy-dispersive X-ray spectroscopy (EDS) elemental spectrum and the corresponding elemental mapping of C/N-ZnO-500. The spectrum displayed the presence of C, N, Zn, and O elements in C/N-ZnO-500, with a uniform distribution. In addition, it can be seen that the content of element C is significantly high compared to the other elements, which is due to the presence of C in the conductive adhesive, in addition to the doping of element C in the generated ZnO. This suggests that, during the phase of transformation from ZIF-8 to ZnO during high-temperature calcination, elements C and N are also doped into ZnO.

**Figure 6:**
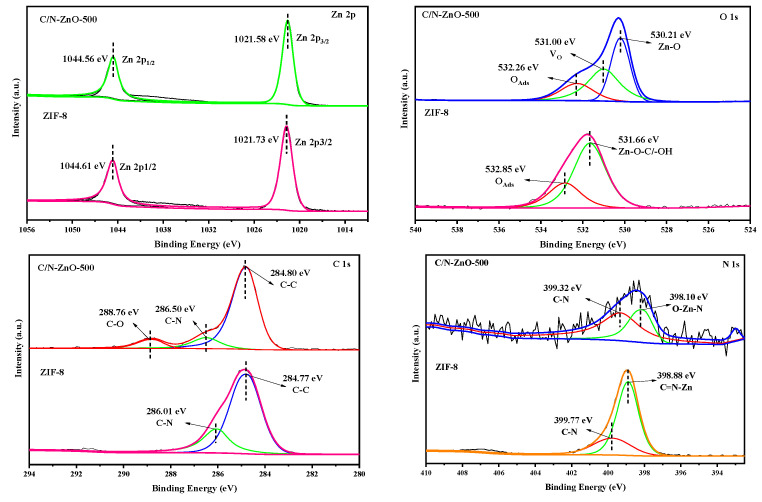
High-resolution XPS spectra of XPSZIF-8 and C/N-ZnO-500.

Figure 6 shows the high-resolution X-ray photoelectrons spectroscopy (XPS) spectra of ZIF-8 and C/N-ZnO-500. The Zn 2p spectra of both ZIF-8 and C/N-ZnO-500 were fitted to two typical characteristic peaks, corresponding to Zn^2+^. The O1s spectra of ZIF-8 revealed peaks associated with Zn-O-C/-OH at 531.66 eV and adsorbed oxygen at 532.85 eV. Conversely, the O1s spectrum of C/N-ZnO-500 exhibited three peaks: Zn-O at 530.21 eV, an oxygen vacancy (V_O_) at 531.00 eV, and adsorbed oxygen at 532.85 eV [26,27]. The C1s spectrum of ZIF-8 displayed peaks for amorphous carbon at 284.77 eV and C-N at 286.1 eV. Conversely, the C1s spectrum of C/N-ZnO-500 showed three characteristic peaks: amorphous carbon at 284.8 eV, C-N bond at 286.50 eV, and C-O bond at 288.76 eV, confirming the successful doping of carbon into ZnO. The N1s spectrum of ZIF-8 exhibited peaks corresponding to the C=N-Zn bond at 398.88 eV and the N-C bond at 399.77 eV. In contrast, C/N-ZnO-500 displayed peaks corresponding to the O-Zn-N bond at 398.88 eV and the N-C bond at 399.32 eV, substantiating the successful incorporation of carbon into ZnO [28,29].

**Figure 7:**
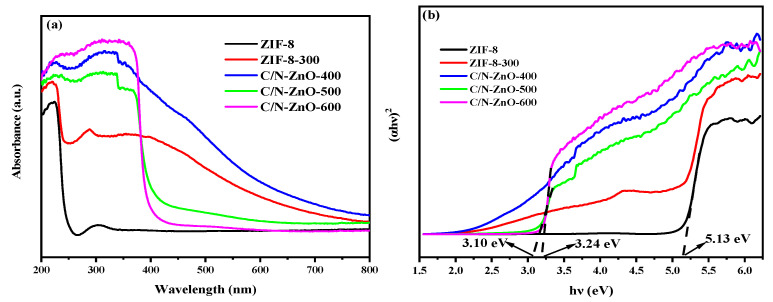
(**a**) UV-visible diffuse reflectance absorption spectrum; (**b**) fitted band gap spectra.

Figure 7a shows the ultraviolet (UV)-visible diffuse reflectance absorption spectra of the photocatalysts. ZIF-8 is predominantly UV-absorbing, with a small visible absorption range and a distinct characteristic light absorption band edge at about 250 nm. Compared to the ZIF-8 and ZIF-8-300 samples, the light absorption range of the C/N-ZnO-x series samples changed significantly. At temperatures of 300 °C and 400 °C, the light absorption peaks show a transition state due to the decomposition of ZIF-8 and the incomplete generation of ZnO, and strong light absorption occurs in the region of 400–500 nm. At temperatures of 500 °C and 600 °C, the decomposition of ZIF-8 is complete, and the light absorption consists entirely of the characteristic peaks of ZnO. Due to the doping of elements C and N and the presence of oxygen vacancies (V_O_), the range of the light absorption in the C/N-ZnO-x series of samples changes significantly from ultraviolet to visible light in the range of the responsive absorption. Among them, the C/N-ZnO-400 sample has the largest visible light absorption intensity. From the band gap spectrum fitted in Figure 7b, it can be seen that the band gaps of the generated ZnO changed significantly with different calcination temperatures; the band gap of ZIF-8 was 5.13 eV, while that of C/N-ZnO-500 was 3.10 eV, which suggests that the doping of the C and N elements, as well as the presence of the oxygen vacancies (V_O_), can regulate the energy band structure of ZnO, which is favourable for improving the light absorption range and enhancing photocatalytic performance [30,31].

**Figure 8:**
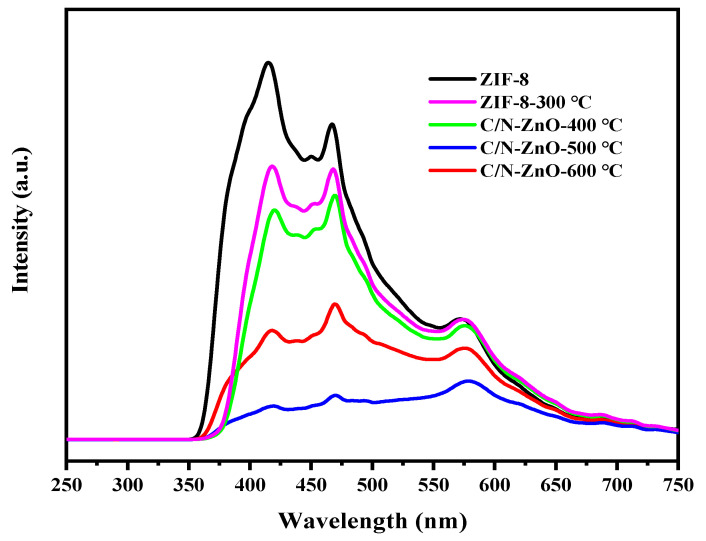
Photoluminescence spectra of ZIF-8 and C/N-ZnO-x samples.

Figure 8 shows the photoluminescence spectra of the prepared samples. It can be seen that the emission peaks of the ZIF-8 and ZIF-8-300 samples have higher intensities, which indicates that their photogenerated carrier recombination rates are fast whereas, in the C/N-ZnO-x series of samples, the emission peaks of ZnO are usually divided into near-ultraviolet and visible emission regions, where the emission peaks at about 390–400 nm are due to the intrinsic luminescence process generated by the jump of electrons from the conduction band to the valence band in their excited state. Luminescence in the visible region at about 430–500 nm is due to oxygen vacancy (V_O_) or zinc vacancy (V_zn_) defects, with oxygen vacancies being the most common cause, which can form localised states of free excitons and cause blue-light emission at room temperature. The emission peaks at about 500~550 nm are usually attributed to the presence of zinc interstitials. From the transformation of ZIF-8 to ZnO, it can be seen that the intensity of the emission peaks of the oxygen vacancies gradually increases compared to the other defect peaks, and the intensity of the photoluminescence peaks decreases and then increases with the increase in calcination temperature. The C/N-ZnO-500 sample has the lowest intensity of the excitation peaks, which suggests that it has the lowest photogenerated electron–hole complexation rate, which is attributed to the doping of the C and N elements and the synergistic effect of the presence of oxygen vacancies (V_O_) and the change in morphology and size. The synergistic effect of the presence of the C and N elements and oxygen vacancies (V_O_) and the change of morphology size, as well as the lower photogenerated electron–hole complexation efficiency, is favourable for the improvement of photocatalytic performance [8].

**Figure 9:**
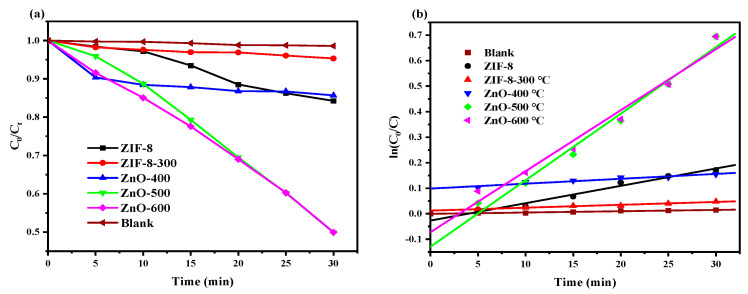
(**a**) Photocatalytic degradation of MO; (**b**) the fitted first-order kinetic curve.

Figure 9a shows the photocatalytic degradation of methyl orange (MO) by ZIF-8 and C/N-ZnO-x under simulated visible light. The photocatalytic degradation efficiencies of the ZIF-8 and ZIF-8-300 samples were very low within 30 min, while the photocatalytic rates of the C/N-ZnO-x series of samples were significantly enhanced compared with those of the ZIF-8 and ZIF-8-300 samples, in which the C/N-ZnO-500 possessed the largest photocatalytic degradation efficiency, with a degradation efficiency of 55%. The enhanced photocatalytic performance is mainly attributed to the synergistic effect of a certain amount of C- and N-doping, the presence of oxygen vacancies, and a large specific surface area, which is found to follow a first-order kinetic curve equation by fitting the photocatalytic degradation process, in which C/N-ZnO-500 has the largest slope, which also indicates that it has the largest photocatalytic degradation efficiency.

**Figure 10:**
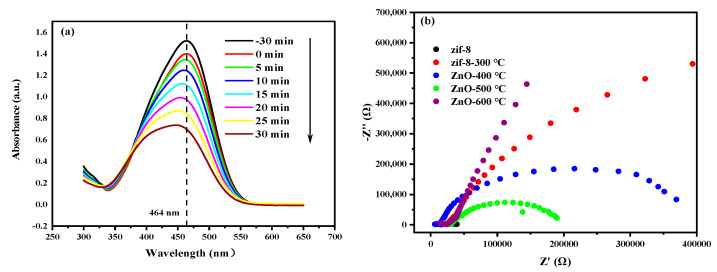
(**a**) Curve of the concentration of MO solution with time; (**b**) electrochemical impedance during photocatalytic degradation by ZnO-500.

Figure 10a shows the variation curve of the light absorption of the MO solution with time during the photocatalytic degradation over C/N-ZnO-500. The MO solution has a typical characteristic absorption peak at 464 nm and, with the prolongation of the photocatalytic time, the light absorption intensity of the MO solution gradually decreases and the concentration of MO gradually decreases, which indicates that the molecules of the MO solution decompose through a chemical reaction with the active groups during the photocatalytic process. Figure 10b shows the electrochemical impedance curves of the prepared samples. Except for ZIF-8, C/N-ZnO-500 has the minimum curvature arc radius, which suggests that C/N-ZnO-500 has the smallest impedance value among the C/N-ZnO-x series of samples, which also suggests that it has the minimum photogenerated carrier mobility, which is conducive to the enhancement of photocatalytic efficiency.

**Figure 11:**
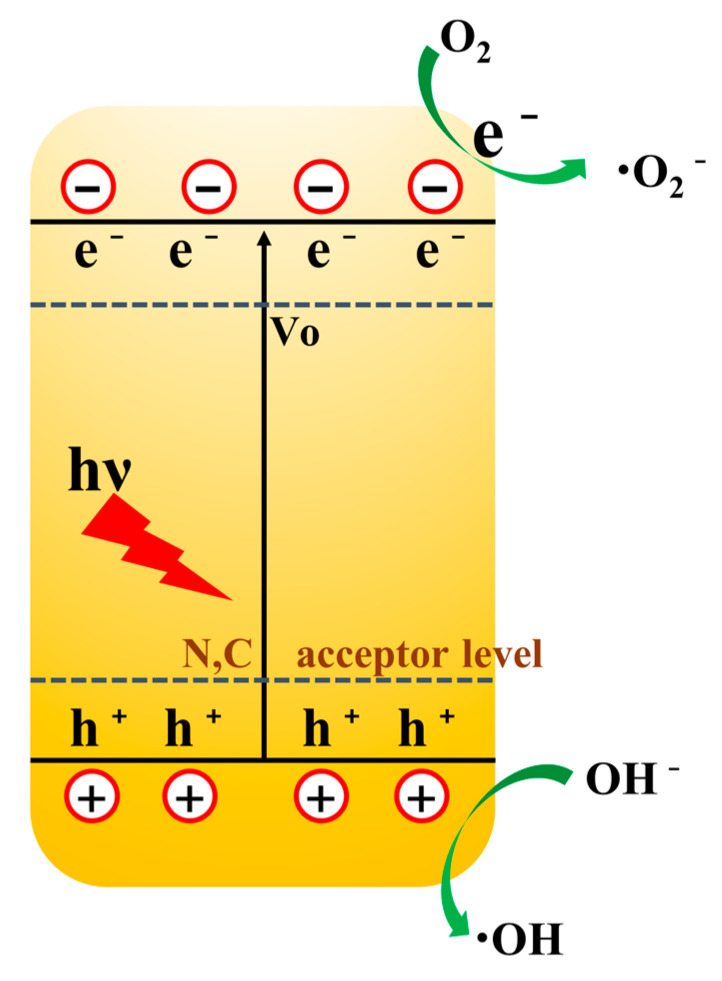
Possible mechanism of photocatalytic degradation.

Figure 11 shows the mechanism of the photocatalytic degradation of the MO solution for the CN-ZnO-x sample. The photocatalyst absorbs photons, and the electrons in the valence band become excited and jump to the conduction band while holes are left in the valence band, forming a photogenerated electron–hole pair, a part of which migrates to the catalyst surface where the electrons undergo a reduction reaction with oxygen and the holes undergo an oxidation reaction with hydroxide ions. The elements C and N, as the acceptor energy levels, are able to capture electrons jumping from the valence band [32,33], which facilitates the separation of the photogenerated electrons, and the (V_O_) of the oxygen vacancy as the donor energy level is able to release electrons to make them jump to the conduction band and increase the carrier concentration. The separation of photogenerated electron holes and the increase in carrier concentration are both favourable to the photocatalytic reaction’s efficiency [34,35]. The doping of C and N elements and the synergistic effect of oxygen vacancies (V_O_) can not only regulate the energy band structure and enhance the absorption of visible light, but also facilitate the separation of photogenerated electron–hole pairs and increase the concentration of photogenerated carriers. In addition, with the special morphology of the MOF precursor, it can provide a large specific surface area for the C/N-ZnO material, which provides more active sites for the degradation and is conducive to the improvement of the photocatalytic reaction rate.

## 4. Conclusions

(1) Metal–organic frameworks (MOFs) were converted into C- and N-co-doped ZnO nanostructures through a high-temperature calcination method, and the material exhibited excellent photocatalytic activity. The photocatalyst was shown to remove 50% of methyl orange (MO) under simulated sunlight irradiation, and the C/N-ZnO-500 sample with optimal photocatalytic performance was enhanced by 50% and 35%, respectively, compared with the blank control and ZIF-8 samples, inspiring the use of MOF derivatisation strategies to optimise the structure and performance of semiconductor photocatalysts.
(2) This study utilised the MOF itself as a C and N source for efficient doping. While conventional doping techniques often require additional C or N sources, the unique structure of the MOF provides a novel direct doping route. In addition, the unique structure of the MOF material provides a large specific surface for the synthesised ZnO, which effectively extends the light absorption range of ZnO and significantly enhances its photogenerated carrier separation efficiency.
(3) The improved photocatalytic performance exhibited by C- and N-co-doped ZnO is attributed to several factors: Firstly, the co-doping of carbon and nitrogen reduces the band gap of ZnO, allowing the material to absorb more visible light. Secondly, the impurity energy levels introduced by doping help the separation and transport of photogenerated carriers. Finally, the MOF-derived nanostructures provide more active sites and a larger specific surface area, further enhancing the photocatalytic performance of the materials.
(4) Based on the findings of this study, future work could focus on optimising the preparation process to achieve further enhancement of the photocatalyst performance, such as regulating the doping level and the porosity of the nanostructures by controlling the calcination temperature. In addition, the C- and N-co-doping strategy identified in this study is expected to be applied to other types of photocatalytic materials, offering the possibility of developing a new generation of high-performance photocatalysts. Meanwhile, more in-depth mechanistic studies will also help to clarify the specific effects of doping on photogenerated carrier dynamics and provide theoretical guidance for the design of efficient photocatalytic systems.
(5) The photocatalytic study of MOF-derived C- and N-co-doped ZnO nanomaterials not only expands the application of MOFs in the field of environmental remediation, but also provides a highly efficient visible-light-responsive photocatalytic system which offers a new way of thinking to solve the problems of energy shortage and environmental pollution.

## Data Availability

Data are contained within the article.
